# Mitral valve endocarditis in an IV ethnobotanical drug user coinfected with HIV-HCV

**DOI:** 10.1186/1471-2334-14-S7-P92

**Published:** 2014-10-15

**Authors:** Ionut Popa, Simona Erscoiu, Aurelia Bumbu, Olivia Burcos, Cristina Pătru, Tatiana Stoicev, Mihaela Ion, Delia Stanciu, Cornel Popescu, Ruxandra Borindel, Vlad Iliescu, Sorin Maximeasa, Emanoil Ceausu

**Affiliations:** 1 Clinical Hospital of Infectious and Tropical Diseases “Dr. Victor Babeş”, Bucharest, Romania; 2 Carol Davila University of Medicine and Pharmacy, Bucharest, Romania; 3 Institute for Cardiac Surgery CC Iliescu, Bucharest, Romania

## Background

Intravenous ethnobotanical drug usage (“legal drugs”) has increased dramatically in the past few years in our country, developing specific pathology characterized by HIV and HCV infections, staphylococcal sepsis, pulmonary or disseminated tuberculosis and neuropsychiatric issues related to the addiction syndrome.

## Case report

We present the case of a young male, MMA, 30 years of age, iv ethnobotanical drug user since 2010 (ex-heroine user since 1994), who was admitted in our intensive care unit for stage III coma, recently installed after prolonged febrile syndrome at home (previously has refused hospitalization). His HIV and HCV tests were positive, chest x-ray was conclusive with staphylococcal bronchopneumonia, cardiac echography showed gigantic mitral valve vegetation and brain MRI showed multiple cerebral abscesses. Blood culture was positive for methicillin resistant *Staphylococcus aureus*, establishing diagnosis of sepsis with multiple system organ failure. He was intubated and received broad spectrum antibiotics for more than 3 months, as well as supportive therapy. He also receives tuberculostatic regimen (because of persistent fever and epidemiological risks) which was stopped when no bacteriological evidences for tuberculosis were obtained. Because immunological suppression was severe (initial CD4 count of 131 cells/mL), antiretroviral treatment with raltegravir and abacavir/lamivudine was given with appropriate psychological counseling. After slightly improvement patient presented sudden chest pain, aphasia and right hemiparesis due to septic emboli from the mitral vegetation. After recovered once again, nine months from the initial admission, having no evidences of active infection, patient underwent mitral valve replacement with good post operator recovery.

## Conclusion

1. Even if less frequently found in drug users, left heart endocarditis is more life threatening, requesting prolonged therapy and high costs. 2. We expected that in the near future this cases to be more and more frequent, because the usage of intravenous drug is not properly controlled, with high risk for spreading HIV, HCV and TB infections in the young generation.

## Consent

Written informed consent was obtained from the patient for publication of this Case report and any accompanying images. A copy of the written consent is available for review by the Editor of this journal.

